# Co-designing a remote rehabilitation tool for Parkinson’s disease: exploratory values and challenges

**DOI:** 10.1186/s12883-021-02519-8

**Published:** 2021-12-16

**Authors:** Patricia Abril-Jiménez, Beatriz Merino-Barbancho, Cecilia Vera-Muñoz, María Teresa Arredondo Waldmeyer

**Affiliations:** Universidad Politécnica de Madrid-Life Supporting Technologies Research Group, ETSIT, Avda Complutense 30, D108, 28040 Madrid, Spain

**Keywords:** Cognitive games, Physical rehabilitation, Physiotherapy, Parkinson’s disease, Wearable system, Co-design

## Abstract

**Background:**

Impaired motor function is one of the early symptoms shown in patients with Parkinson Disease (PD). For this reason, rehabilitative interventions have been used for many years to improve motor and non-motor symptoms. Among them, the use of music therapy has shown benefits in helping to overcome some of the most common motor dysfunction. Addressing the challenge of providing access to this type of therapy, this document presents the collaborative design process to develop a remote training support tool for PD based on music therapy.

**Methods:**

A qualitative study with creative co-design methods was used in which different groups of healthcare professionals, patients, and relatives participated in six iterative sessions. Workshops were designed and structured to incrementally discover requirements and needs and validate the proposed prototype ideas.

**Results:**

The study provided key aspects that were used for the development and validation of the proposed prototypes for the remote music-based training support tool for PD. Up to 20 factors that had a positive and/or negative influence on patient access to training were detected. These factors were classified into three common themes: daily activities and independence, participation in treatment and barriers to daily treatment, and self-management and personalization of information and telecommunication technologies (ICT).

**Conclusions:**

This paper shows the results of a collaborative design process aimed at identifying the different factors, relevant to patients with PD, to improve their access to remote ICT-based training therapy and their expectations regarding alternative therapies, such as music. The participatory design methods and the iterative model used helped overcome many of the traditionally barriers that this type of technological support solutions usually have, facilitating the future participation.

## Background

Parkinson’s disease (PD) is the second most common neurodegenerative disease after Alzheimer’s disease, affecting more than 10 million people worldwide [1]. People living with Parkinson’s suffer from motor and non-motor symptoms that affect their daily activities, notably reducing their quality of life (QoL). Motor symptoms affect movement and include tremor, rigidity, slow movement, freezing, falls and dizziness, muscle cramps and dystonia as the most common manifestations, as well as other common expressions directly related to pharmacological treatments such as dyskinesia and akinesia [2]. Nonmotor symptoms affect, among others, pain, mental health, speech, and communication [3].

Although it currently does not have a cure, there are several therapy options available to slow the progression of PD and treat its symptoms, with pharmaceutical and / or surgical interventions being the most common treatments [4]. However, in conjunction with these interventions, non-pharmacological therapies that involve physical activity and training, such as physical therapy and occupational therapy, have been shown to be useful tools to address the motor and non-motor symptoms of Parkinson’s disease [5, 6]. These therapies complement pharmacological and medical treatment and focus on improving the physical capacity of the patient, as well as the physical and cognitive functional abilities. This has a positive effect on the disease management and helps to improve the patient quality of life, safety, and independence in performing daily activities. As an example, physiotherapy has shown its effectiveness in improving one of the most debilitating symptoms of PD, freezing of gait, which involves the sudden inability of a person to walk despite her intention to do so [7, 8].

Similarly, the use of music therapy and external rhythmic cueing (ERC) combined with conventional therapies has been shown to be effective for motor training in PD with positive effects on the quality of life of patients, alleviating symptoms such as rigidity, postural instability, or gait-related problems, and side effects of the medication such as bradykinesia [9, 10]. The justification of these positive effects can be explained in several ways. One of the proposed mechanisms is that therapies based on music rhythms and signals activate some specific neuronal networks that are usually healthy for longer periods in the brain of a patient with PD (i.e., cerebellar thalamocortical networks), which are different from those used to perform self-generated movements (i.e., basal ganglia thalamocortical networks) that are disrupted in PD [11]. These networks would act as a compensatory mechanism capable of enhancing motor behaviour in PD, allowing patients to better synchronize their movements, move faster and walk at a regular pace, even with longer steps [12]. Furthermore, training using auditory signals provides benefits not only for motor symptoms, but also for nonmotor ones, such as those associated with cognitive functioning [13, 14], because the provision of rhythmic timed cues allows the brain to anticipate movements, with an improvement in their precision and synchronization [11]. Although the characteristics (type, frequency, duration and intensity) of these non-pharmacological interventions to optimize impact and effectively address PD are not clear yet, there is an increasing evidence of their positive effect on the treatment of this disease [8, 15], as well as on their influence on slowing down its progression [16–18]. Furthermore, several studies suggest that the effect of this type of tasks-based and context related training could increase the ability of patients with PD to perform meaningful tasks in their daily life, assuming it is performed in familiar environments, such as home [19]. Nevertheless, the effect of physical training has been shown to decrease after the intervention is complete, which suggests that continuous lifelong training is needed for these patients [8, 15, 18].

To address the need to provide daily access to previously presented therapeutic modality as complementary interventions for patients with PD by providing a remote training intervention,

This paper presents the design process of an information and communications technology (ICT) solution intended to address the previously presented needs and to provide PD patients with a remote rehabilitation intervention based on the use of music therapy and ERC that would complement their standard treatment. Such a solution was conceived to offer training in the user environment, anywhere and anytime, providing a set of different activities adapted from current physiological practice, including motor, sensorimotor and cognitive exercises accompanied by music, rhythmic cues, and a metronome.

The envisioned solution included a smartphone application and a pair of sensors to be placed on the wrist or ankle user depending on the exercise to be performed. The application was linked to a web platform meant for therapists, where they can monitor the progress of remote training sessions and recommend some concrete exercises. The final objective of the solution was to bring patients and therapists together in remote rehabilitation sessions, seeking to provide lifelong treatment and improve disease follow-up.

As part of the design process, this article describes a qualitative study aimed at discovering and obtaining the requirements of the described solution to support its design and development. The study involved different types of stakeholders, including medical professionals (i.e., neurologists), therapists (i.e., physiotherapists, music therapists, occupational therapists), and end users (i.e., people with PD).

## Methods

This section describes the methodology used to discover the key elements necessary to ensure the acceptability, usability, and usefulness of a remote training ICT-based solution to support patients with PD on their daily training needs, and the interest of PD patients and therapists in incorporating music therapy and ERC into the system. The selected method was a qualitative study that counted with the participation of professionals, stakeholders, patients, and relatives and was designed to identify the requirements, as well as the crucial functional elements for the follow-up of patients that a solution, such as the proposed one, should have.

### Study design and procedures

The study aimed to explore and discover the rehabilitation and training needs of patients with PD, as well as the needs and requirements of their therapists and relatives. These would be later used to design a system, as a useful solution for daily remote training of PD. With this objective, a series of co-creation workshops were planned, with a maximum duration of 3 h each and the following structure.

After a short welcome, the involved research team and participants were briefly introduced, together with an explanation of the purpose and nature of the session. This introduction included an interactive short presentation of the first version of a mobile application prototype [20], developed before conducting the study. During the demonstration, the functionalities of the rehabilitation program (both the already defined and the new proposed ones) as well as the acceptance and usability of the proposed solution were discussed with the participants in the workshop. This discussion was driven by a guide, prepared by the research team prior to each workshop, that integrated a set of components to assist the debate among participants. This guide was updated after each session and considered the results of previous workshops and the characteristics of the participants.

During the different workshops, the activities included experimental observation, journaling exercises, conversations, and storytelling. The selection of activities was made based on the profile of the participants and the specific objective of the workshop, with the aim of incrementally discovering the unmet needs, functionalities, and accessibility and usability barriers that would support the design and development of the training application. Nominal group techniques [21] were introduced to prioritize the information collected, while focus group techniques and semi-structured interviews were used to reflect the perceptions of the participants, and to understand the daily training requirements of the patients when using rehabilitation facilities and how to adapt these training activities to the proposed solution.

The focus groups were designed using a funnel approach consisting of a series of questions aimed at discovering the routines of the participants during the rehabilitation sessions and identifying the measuring indicators and barriers to remote rehabilitation. Semi-structured interviews were conducted to discover the perceptions of the participants when using the solution prototype, once it was available. In this case, the prototype was shown in the session before starting the discussion.

**Fig. 1:**
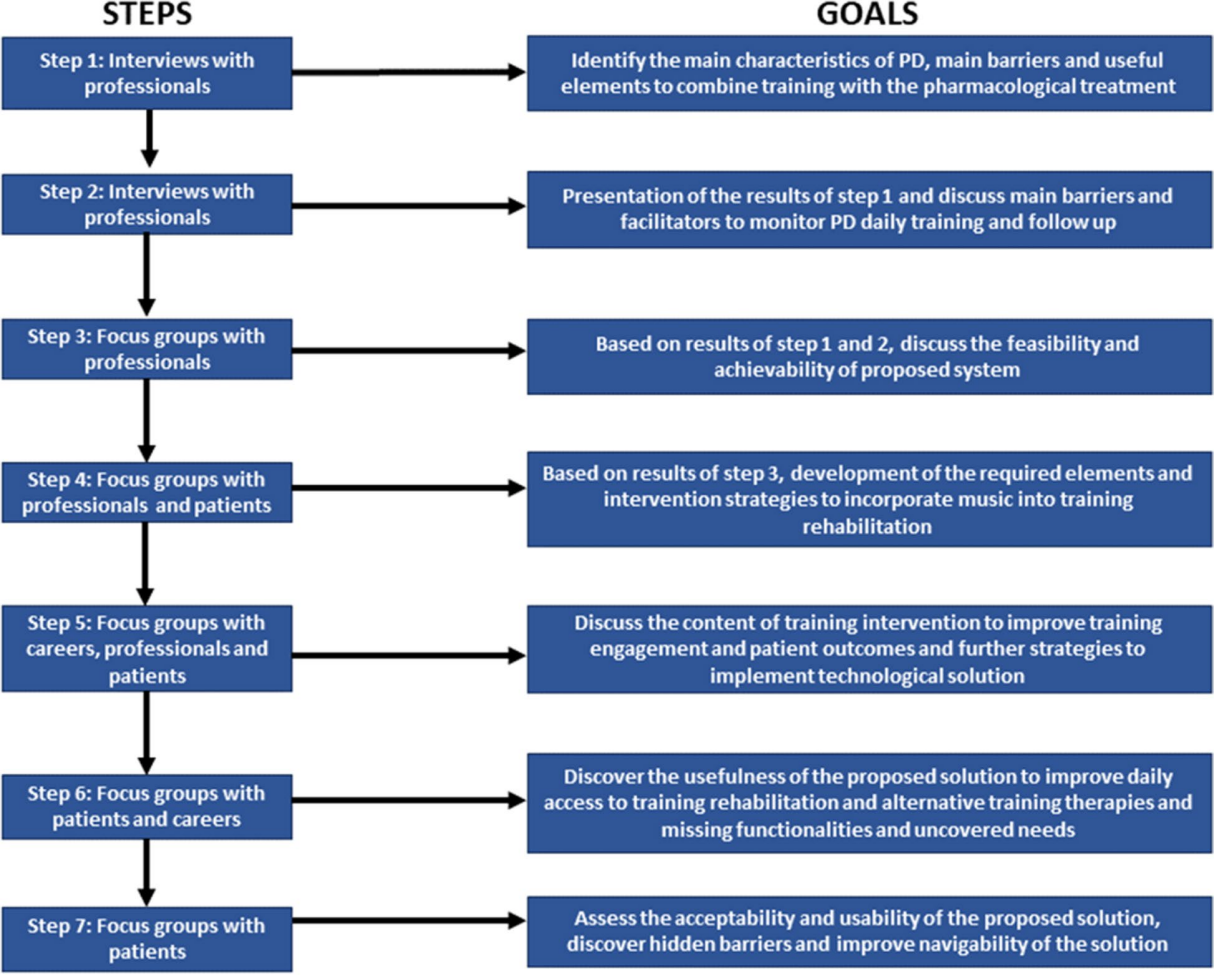
Flow chart of the different steps of the codesign methodology and their specific goals

Both the semi-structured interviews and the focus groups were designed to be open-ended, allowing flexibility to explore the themes and solutions that were presented in each of the sessions. Figure 1 shows the different steps in the presented process and the particular goals in each case.

### Setting and participants

A total of seven workshops, aimed at exploring the rehabilitation needs of PD, were organized between December 2017 and January 2019. The composition of the workshops varied in each session, to ensure a fluent group dynamics, deep interactions, and diversity between them, in terms of background, sociocultural characteristics, and disease understanding. The characteristics of the session, duration, materials, and discussion dynamics were also adapted to improve the needs discovery process. The adapted processes of group diversity and creativity facilitated spontaneous finding of themes, topics, and ideas based on divergent views and complexities of the topic.

**Fig. 2:**
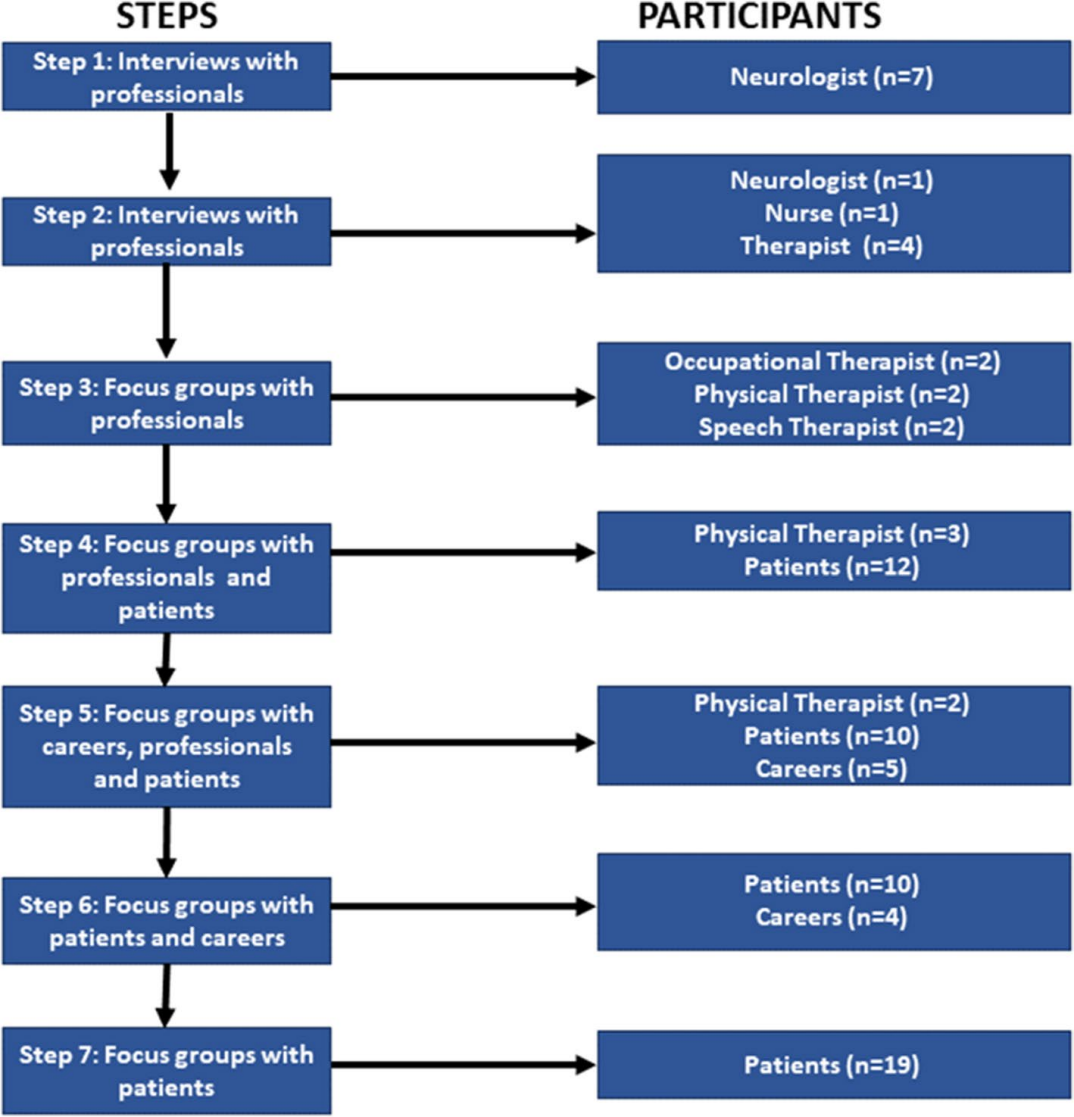
An overview of the participants in all steps of the investigation

In total, 84 people participated in the workshops (24 professionals, 9 caregivers and 51 people with PD: Hoehn and Yahr scores from I to IV). They were recruited from different Parkinson’s associations, rehabilitation centres, and hospitals in Spain. Figure 2 describes the participation of each type of user in the different steps of the study, Table 1 describes the main characteristics of the participants needed to configure the different sessions, while Table 2 presents more detailed characteristics of the participants in the subgroup of patients.

**Table 1:** General description of the participants characteristics

Participant group	Total number	Gender (female (%) / male (%)
Neurologist	7	0%/100%
Nurses	1	100%/0%
Therapist		
Occupational therapist	4	100%/0%
Physical therapist	9	66,66%/33,33%
Speech therapist	2	100%/0%
Caregivers		
Couple	6	66,66%/33,33%
Children	3	66,66%/33,33%
Patients	51	39,21%/60,78%

**Table 2:** Patients involved age and PD general characteristics

Patients Characteristic	N (%)
Age	
40–45	9 (17,64%)
46–50	9 (17,64%)
51–55	9 (17,64%)
56–60	8 (15,68%)
61–65	7 (13,72%)
66–70	5 (9,80%)
Over 70	4 (7,84%)
Hoehn and Yahr scores	
I	16 (31,37%)
II	14 (27,45%)
III	13 (25,49%)
IV	8 (15,69%)
Years of Disease progression	
Less than 5	17 (33,33%)
5–10	18 (35,29%)
10–15	9 (17,64)
More than 15	7 (13,72%)

The first workshop (*n* = 7) tried to discover the main characteristics of PD treatment and the influence of physical training on pharmacological treatment. The following two workshops (*n* = 6 each) aimed to better understand the components of the rehabilitation session, the daily routines and the environmental conditions of the training, and how external factors, such as music, could influence the performance of the training of patients and the standardized indicators used by professionals. These first sessions counted with the participation of therapists that provided their work perceptions and real experiences they had had dealt with in work conditions. In addition, the individual patient’s needs were explored to determine training follow-up indicators to be measured with the solution prototype.

During the fourth (*n* = 15) and fifth (*n* = 17) workshops, PD patients were incorporated. The main objective of these two workshops was to understand the process of engaging with the training and the main barriers for the patients to practice on their own at home. The final two workshops, sixth and seventh (*n* = 14 and *n* = 19, respectively), were aimed to evaluate a functional prototype of the solution, consisting of a mobile app on a smartphone, a couple of inertial sensors to monitor exercises, and two web applications for professional follow-up and patient self-management. These final workshops were multidisciplinary, with the participation of professionals, therapists/caregivers, and individuals with PD.

Before conducting the qualitative study, a protocol was prepared and approved by the Research Ethics Committee of the Universidad Politécnica de Madrid (UPM). All study participants signed the consent forms after being informed of the participation conditions and before starting their participation in the workshops.

### Process for data collection

During the study, all data was collected through interviews and focus groups. The whole process had different interconnected steps, starting with interviews (in the first sessions), which provided a first identification of barriers, facilitators, and useful elements to apply digital solutions to train daily routines in patients with PD. The process continued with focus groups (in the latter sessions), in which the results of previous interviews were presented to participants along with some possible related digital solutions to solve or overcome the problems encountered during the training process. These focus groups involved discussions about the feasibility and applicability of the solutions presented to the daily routines of patients with PD.

#### Interviews

The interview questions were designed to develop a comprehensive framework to better understand the daily training needs of PD patients and their families, while discovering the working processes during rehabilitation sessions. The interviewers were supported by an interview guide to ensure that all relevant information was obtained. This guide was prepared in advance by a multidisciplinary research team that included usability designers and health professionals and considered the results from previous collaborative sessions (when available) and the unmet functional and non-functional requirements for the future remote rehabilitation system. The interviews were conducted in Spanish and lasted approximately 1 h. Individual interviews were recorded with the consent of the interviewees, and the audio records were later verbatim transcribed for data analysis. Additionally, the interviewees took notes about the nonverbal aptitudes of the participants during the interview.

#### Focus groups

Different focus groups were planned, with the participation of at least six participants in each of them, to explore and discuss the main results of the interviews and previous focus group sessions and iteratively design a solution that addressed the needs of patients and professionals in terms of rehabilitation training.

These sessions comprised the signature of the informed consent of the participants, a short presentation of the focus group objectives, and a discussion of the main topics derived from the previous sessions, moderated by the research team. In this case, informed consent included a short questionnaire to collect the sociodemographic information from the participants.

### Data analysis

The digital recordings were verbatim transcribed. These transcriptions were reviewed and analysed by five members of the research team and coded according to thematic content analysis using Atlas.ti software [13] version 8. To bring a wide variety of knowledge and vision to the analysis process, the team comprised researchers with different backgrounds, including two computer science specialists, two usability and accessibility experts, and an active and healthy ageing expert. The team members read all the material collected to identify the main discussion topics, content, and main findings. Data saturation was considered when no new topics were identified.

## Results

Th interviews and focus groups with participants revealed several topics and factors of daily rehabilitation training programs, including some access problems. Iterative and incremental knowledge gathering about these factors during the study helped design an effective technological-based training program and improve the access of PD patients to daily rehabilitation.

### Daily activities and independent living

During the workshops, one of the main concerns of younger participants with PD was maintaining their independence, while the main concerns of older patients were the need for institutionalization and the excessive burden of their relatives due to their lack of independence. In both cases, the burden caused by their disease was a major concern.

There was a strong relationship between the burden and patients’ physical and emotional functional status.

*‘She’s seen me deteriorate … I can feel her worries... and it will no doubt worsen.’* (Patient, 63 years old).

Daily access to rehabilitation had positive effects on the main identified areas of patient needs and barriers to care, such as symptoms management and wellness strategies. However, access to daily rehabilitation therapy was not easy for all the participants for several reasons. The most common were the difficulty for traveling to the rehabilitation centre and the time spent on the entire trip, including travel time and rehabilitation sessions.

*‘I feel tired walking, I cannot drive, so it is very hard to go every week to the training centre... I spend more than 2 h to go and back’* (Patient, 47 years old).

Therefore, an adaptable remote training program was perceived as a key instrument for the daily management of the illness and, especially, to maintain independence longer. Consequently, exercising on their own was appreciated by some affected participants, especially those in the early stages of the disease, who reported increased distress after attending a conjoined therapy session because they could realize what it would be like when their disease progressed. This was supported by some of the therapists who considered it was better to have people with similar abilities and disease progression in the same group, although the lack of resources made this very difficult.

*‘Perception of diseases is different from one another. I see patients who become anxious when they see patients with poor mobility, they get depressed, and they feel disappointed in the usefulness of rehabilitation.’* (Therapist 1).

Together with physical decline, the progressive loss of cognitive function was one of the main concerns of the early affected participants, and they considered that this type of therapy was a mismatch of available resources. Among all related complications associated with cognitive decline, attention, memory, and problem solving were the main identified problems during the workshops. The introduction of early detection strategies and cognitive training strategies to delay this secondary effect of PD was very welcome among participants, especially by social therapists’ and mid-age patients.

During the first workshop, physical training was perceived as positive for reducing cognitive impairment by both neurologists and therapists. This assessment was also supported during subsequent workshops, where patients reported that coordination training helped them to better deal with complex daily activities, such as those that needed multitasking. Therefore, some therapists and participants argued, when asked about alternative therapies to pharmacological treatment, that daily coordination training sessions, such as those supported by music, made their body move in new ways and promoted the use of their brains.

*‘Medication is important but not enough. Rehabilitation is also important, and you need to involve them to influence their quality of life’ (Therapist 4).*

For these reasons, they felt that this exercise effort could be translated into other areas of their daily life, such as doing homework, planning, or cooking.

Finally, the participants appreciated the possibility of combining physical training with cognitive exercises, especially if the exercises were serious games that also provided them with entertainment.

*‘I have stopped to see my friends, because I get tired after training, but it would be great if I could combine leisure and treatment’* (Patient 43, 49 years old).

The cost of the service, the lack of public places in Spain, and the waiting lists were big problems for most of the participants. The combination of these three elements was perceived as the main for accessing these alternative therapies, which were considered crucial for the treatment of the disease. Participants, both professionals and affected patients and their families recognized that rehabilitation was the best option, together with medication, to keep symptoms under control and minimize their impact on daily activities.

*‘I was on a waiting list for six months to a year to see a neurosurgeon and six months more to see a rehabilitation specialist. I cannot pay for a private rehabilitation session … so much cost’* (Patient 3, 56 years old).

Th participants demanded more communication with their therapist so they could overcome daily doubts or small problems when they were working alone at home. In this sense, continuous follow-up and self-management of their rehabilitation goals and expectations were appreciated among younger participants with PD and some of their caregivers. The suggestion of having a personalized dashboard where they could visualize their achievements, the next training sessions, or the pending exercises was appreciated.

*‘I think (personalized dashboard) would participate more in treatment if I felt supported, just if I have doubts or I have new symptoms’* (Patient 32, 49 years old).

*‘That (personalized dashboard) mitigates the cost of all this, and also saves time. Furthermore, I cannot be sure that my father does exercise’* (Caregiver, 53 years old).

### The need for training engagement and daily treatment barriers

Among the treatment services, neurology, physical and language therapies were identified as the most important ones. Therefore, both professionals and patients agreed that a system such as the proposed one should be useful to facilitate the automatic generation of a report on Instrumental Activities of Daily Living (IADL) and rehabilitation challenges, and thus enable the collaboration between the neurology and rehabilitation centres in treatment. This evaluation of the symptoms or feelings of the patients during the training sessions was recognized as helpful in identifying issues that could be formulated as goals.

*‘In patient treatment, we have a multidisciplinary team. Sometimes there is a lack of communication, one can see something, the other another. That could be useful’* (Doctor 5).

Neurologists and therapists considered that self-assessment instruments were not always reliable. Therefore, the use of monitoring technologies, such as the proposed solution, which recorded and evaluated performance in real-time exercise, could be useful in identifying problems that could remain hidden during control and follow-up visits. The participants coincided in highlighting that contributing to more fluent coordination and information exchange between people involved in patient care helped patients and their relatives achieve their desired balance.

Patients and therapists of the rehabilitation centres agreed that pharmacological therapy did not solve most of the problems of daily activity for patients, especially those related to muscle stiffness that reduced stretch and affected each of their movements, particularly gait. All the participants recognized the positive effects that non-pharmacological therapy and physical training had on this.

*‘The benefit (of using music) for me is to see that my patient enjoys the exercises when they are in the session, they can forget everything else and focus on their movements”* (Therapist 5).

However, patients identified that some of the common training exercises were repetitive and boring, so it was difficult to engage with them at home and during the exercise on their own. Younger patients participated in collective generic training sessions, not necessarily adapted for PD patients, so they could overcome boring exercises and engage in physical rehabilitation.

*‘My greatest difficulty when training is repetitive up and down movements. I get bored easily. I prefer to try other activities, dance is very funny’* (Patient 7, 69 years old).

Some of the participants and two of the therapists recognized that the use of music helped them to be more engaged during the training session; even the results obtained were better. Most of the participants used music during their exercise sessions, even if they were only walking. Furthermore, these two therapists recognized that they used specific music therapy strategies, not only to engage with their patients, but also because the results were better, and the patients were involved in rehabilitation goals more easily and for longer. This type of therapy was welcomed among patients because it was perceived as more motivational and made them feel a constant support of therapy, which was identified as the main issue in remaining engaged and enrolled in the training programs. Participants indicated that this type of music therapy helped them regain control over their lives, as they perceived it as a way to mitigate their symptoms and had a direct positive direct impact on their mood and attitude towards the disease.

*‘It’s like you’re doing another activity, not just rehabilitation because you’ve got a disease. I feel better. It may help me normalize the disease* (Patient 23, 62 years old).

### ICT, self-management, and personalization

The use of applications and mobile devices for treatment control, monitoring and accessing to information was perceived as a facilitator for treatment follow-up and daily management of the disease. During the workshops, patients and professionals highlighted these benefits.

However, the digital gap was also present among aged patients. Although younger participants were enthusiastic about the possibility of participating in their quest to find ways to mitigate more inconvenience symptoms in their daily lives with the support of new applications and devices to help them cope with challenging situations, older participants considered that monitoring and training support applications could be more useful for their caregivers and relatives, as they could better follow them up. Some of them considered the use of wearables and sensors very intrusive and did not expect to use this kind of device, even if they could support their training and treatment management at home.

*‘I like to take the best out of everything, and technology is there. It would be great to enjoy new activities thanks to this”* (Patient 43, 45 years old).

*‘I cannot use a smartphone too much to learn, but maybe my sister finds it useful’* (Patient 8, 76 years old).

Guidance and flexibility to adjust content and structure to meet individual needs were appreciated in such rehabilitation and monitoring support applications and tools. Both patients and professionals highlighted the importance of a customized rehabilitation treatment that could adapt the training to their daily needs and routines. Continuous training at home was greatly appreciated because it contributed to a better understanding of their disease, supported them in dealing with daily routines and increasing disability, and adapted the training to their disease cycles. Positive feedback and self-control of changes in physical ability were highlighted as aspects of increasing patient confidence and motivation to participate in daily training and self-management of the disease.

*‘The most important thing for patients is improving mobility, and exercise should be continuously adapted to maintain motor function as long as possible, this has a strong impact on your quality of life.’* (Doctor 1).

*‘I feel that way (using the proposed solution) I get control of my life. I can do my exercises when I feel better, so I get more benefits from them’* (Patient 32, 58 years),

### Summary of the proposed topics and functionalities

During the different sessions, the participants identified factors and issues that could become barriers (i.e., circumstances that blocked the successful achievement of the patient’s objectives), complicating factors (i.e. daily situation or activity that makes daily activities of the patients more difficult), and facilitator (i.e. an element that helps make a daily activity easier). These factors served to define the potential requirements of a training system. The factors were grouped into three main topics: 1) daily activities and independence addressing the importance of treatment to support the daily situations that PD patients and their caregivers had to face as the disease progressed and how this affected their independence, 2) the need for training, participation, and daily treatment barriers addressing the practical implications of physical activity access and how PD patients and their relatives and medical team participated in their routines, 3) ICT, self-management, and personalization aimed to discover how participants perceived technology and their requirements to get engaged, as well as their expectations. Each of the topics was divided into a set of factors that emerged during the different sessions and whose purpose was to explore the different perspectives of the participants in each of the main topic. Table 3 shows the different topics, the factors associated with each of the topics, and the classification of each factor according to the perception of each of the participant groups (i.e., professionals - which included the neurologist, nurses and therapists-, caregivers, all of them, patients’ relatives, and patients). The following sections describe in detail each of the topics and factors identified.

**Table 3:** Barriers, facilitators, and complicating factors in the development of a remote training rehabilitation system for PD. In case the identified factor was not detected by the group, it is identified as not applicable in the table

Topic	Factors	Professional	Caregiver	Patients
Daily activities and independence	Independent living	Not applicable	Barrier	Complicating factors
	Daily access to rehabilitation	Facilitator	Barrier	Barrier
	Transport and travel	Not applicable	Barrier	Barrier
	Disease acceptance	Complicating factors	Complicating factors	Barrier
	Cognitive function	Facilitator	Complicating factors	Facilitator
	Therapy cost	Not applicable	Barrier	Barrier
	Alternative therapies	Facilitator	Facilitator	Facilitator
	Daily follow-up of treatment achievement	Facilitator	Complicating factors	Barrier/Facilitator
Treatment engagement and daily treatment barriers	IADL support	Facilitator	Complicating factors	Complicating factors
	Reports	Facilitator	Facilitator	Facilitator
	Continuous monitoring	Facilitator	Facilitator	Facilitator
	Training impact on QoL	Facilitator	Facilitator	Facilitator
ICT, self-management, and personalization	Telemonitoring	Facilitator	Facilitator	Facilitator/Barrier
	Personalization	Complicating factors	Facilitator	Facilitator
	Continuous follow up	Facilitator	Facilitator	Facilitator
	Wearables	Facilitator	Not applicable	Facilitator/barriers
	Self-training	Facilitator	Facilitator	Facilitator

During the collaborative sessions, the participants identified the feasible elements and requirements required in a design of a remote training solution based on music therapy for PD. These would be later translated into functionalities. Table 4 maps the previously identified factors as facilitators, barriers or complicating factors with the envisioned functionality/requirement proposed by the participants. Based on this initial list of functionalities/requirements, the feasible elements of the solution were further identified during the study and used later in the system design.

**Table 4:** Training support elements for system design discovered during the sessions mapped with their targeted factor

Targeted factors	Strategies to implement the element in the system
Independent living	•-The content focuses on holistic rehabilitation of cognitive/physical function based on IADL common activities
Daily access to rehabilitation	•-Mobile-based application available in Android/ iOS, and cheap sensors •-Sensors/algorithm are capable of monitoring performance and adapting to the user’s performance. •-Assess performance and deliver reports •-Sensors are easy to turn on/off and place. •-Personalization and scheduling of rehabilitation session
Transport and travel	•-Mobile-based application available in Android/ iOS, and cheap sensors •-Follow the functionalities of the achievements and exercises done (measurements and outcomes)
Disease acceptance	•-Monitoring of achieved goals •-Engaged personalized exercises, adaptable difficulty levels and customizable music
Cognitive function	•-Cognitive games
Therapy cost	•-Mobile-based application available in android/ iOS, and cheap sensors
Alternative therapies	•-Music therapy-based interventions
Daily follow-up of treatment achievement	•-Reports, measurements, and results for easy follow-up of achievements
IADL support	•-Cognitive/physical exercises
Reports	•-Reports, measurements, and outcomes for easy follow up of the achievements
Continuous monitoring	•-Assess performance and deliver reports
Training impact on QoL	•-Monitoring •-Goal achievement
Telemonitoring	•-Possibility to send messages to the professional
Personalization	•-Music and exercises level
Continuous follow up	•-Assess performance and deliver reports
Wearables	•-Cheap Sensors •-Sensors ergonomics •-Easy to power on/off. •-Only capture the need information
Self-training	•-Personalization of level •-Continuous follow up

## Discussion

During the study conducted to collaborative design a remote training ICT based solution to support patients with PD, we used mixed methods to create a comprehensive understanding of the participants’ needs, desires, and barriers when incorporating physical training and rehabilitation as part of the treatment. The results demonstrated that people with PD appreciated the benefits of daily rehabilitation on their quality of life. This improvement in quality of life also benefited the interaction with their social environment and had a positive impact on their relatives and friends. The results obtained are similar as the ones obtained in other analogous studies [22–24].

The goal of the rehabilitation solution codesigned within this study was to provide an integrated approach to remote rehabilitation that supported self-management of training goals for each individual, while providing access to alternative therapies that had better engagement results in the medium term. Although existing training applications and remote training systems did not support real-time feedback and were based on traditional repetitive exercise series [25, 26], there was increasing evidence of the need for a paradigm change that provided key active ingredients to empower PD patients in their daily rehabilitation programs.

The presented study provided information on the strengths, limitations, and barriers of current rehabilitation programs and global care for PD patients, and particularly highlighted the difficulties for accessing daily rehabilitation, which were one of the main concerns and difficulties of the participants, with a clear negative impact on their daily activities and consequently on their perception of quality of life. The lack of public rehabilitation centres and professionals, the increasing need for assistance moving to the rehabilitation centre premises, and consequently the associated cost of rehabilitation were identified as the main barriers to have a daily rehabilitation program. Consequently, the participants welcomed the use of remote training programs and the valued positively the option of working together on the definition of the requirements of this type of solution. In particular, they emphasized the usefulness of systems to provide self-management support that facilitated daily access to a remote training program supervised by their therapists.

However, these dimensions were not the only ones that presented challenges in accessing rehabilitation programs, but also the lack of personalization of these programs themselves. PD affects a wide range of patients, from young people in their 30s or older to older adults with other disabilities due to age [27]. The customization dimension is particularly challenging for younger patients, who demand rehabilitation programs adapted to their physical activity that also serve as leisure time and social participation [28], so that they can keep their symptoms under control. In this sense, they appreciated alternative therapies that combined traditional repetitive rehabilitation movements with music and/or dance. There was identified a need for tools that facilitated self-management, therapist follow-up, and coordination between the care team. These aspects were considered essential for the achievement of training goals.

The results obtained in the study also suggested that rehabilitation programs were not perceived as effective in patients whose impairment fluctuated significantly depending on their emotional status (that is, anxiety, fatigue) or medication, as in those who did not suffer these episodes. In this sense, the possibility of adapting not only the program but also the timing and exercise schedule to those moments could make patients feel more active and improve the rehabilitation results and would have a positive impact on their participation in therapy. Better participation in therapy was associated with better QoL perception, since patients felt that they could maintain their symptoms under control. The study participants considered that synchronization of music therapy and training according to their state had a very positive effect on their physical and emotional state. This had also been demonstrated in several previous research studies, suggesting that music had positive effects in improving pain and balance, but also in improving patient mood [10]. Finally, most of the factors discovered during the sessions were focused on physical training and the influence of alternative therapies, such as music therapy, on the adherence to daily exercise routines and the perceived usefulness in patient quality of life. This could be a consequence of the most obvious motor symptoms being bradykinesia, tremor, and rigidity, and the fact that other motor symptoms directly related to processive muscle weakening and loss of motor control are not evidence in the early stages of the disease [29]. However, there are several aspects related to patient independence and quality of life that could also benefit from the use of music therapy in the early stages of the disease, such as impaired speech as the vocal muscles of patients weaken or reduced facial expression due to a deficit in motor control of the facial muscles [30]. These symptoms have a high long-term impact on the quality of life of patients and their caregivers, as they restrict their normal social relationships and the expression of their feelings. Future work will focus on discovering how the proposed remote training system could combine other art therapies such as choral singing [31], dance movements, and theatre [32] to improve the quality of life of patients with other motor symptoms such as hypomimia, hypophonia, etc.

### Methodological considerations and limitations of the study

In our study, the participants were recruited from different patient associations, hospitals, and rehabilitation centres in Spain, with different levels of affectation and age. The number of participants of the same age, with an additional functional decline due to age, represented the highest number of patients with advanced-diagnosed PD. However, the results could have been influenced by geographic and selection bias, as those who agreed to participate could have been more proactive in their treatment management. The limited geographical area of the study could be a limitation of the study that could make it difficult to transfer its results to areas more dependent on the national health system, such as the availability of rehabilitation resources and their cost.

## Conclusions

Patient experiences are better identified through focus groups [33] because they allow interaction with their pairs, encourage participation, and promote requirements discussions, leading to the discovery of unmet needs, unidentified barriers, etc., that the new proposed solutions can meet. The process followed for the co-design of the ICT-based remote training solution to support PD patients, and in particular the study carried out allowed us to incorporate all the requirements and needs discovered during the different sessions into the solution design, making it acceptable and perceived as useful by patients with PD. The continuous involvement of each stakeholder involved in patient care allowed for an iterative discovery of needs and requirements and an incremental improvement of the design of the solution. More research would be needed to test the proposed solution in real-life settings to assess the effectiveness of the designed solution and examine long-term results.

## Data Availability

The datasets analysed during the current study contain identifying information and are therefore unavailable publicly. Source documents of the research project are securely kept at the Universidad Politécnica de Madrid, Spain. Data can be made available through contacting the last author.

## Abbreviations

ERC: External Rhythmic Cueing
ICT: Information and Communications Technology
IT: Information Technology
PD: Parkinson’s Disease
QoL: Quality of Life
UPM: Universidad Politécnica de Madrid
